# Tunable magnon polaritons via Eddy-current-induced dissipation in metallic-banded YIG spheres

**DOI:** 10.1038/s41598-026-55914-x

**Published:** 2026-08-19

**Authors:** Tatsushi Uno, Shugo Yoshii, Sotaro Mae, Ei Shigematsu, Ryo Ohshima, Yuichiro Ando, Masashi Shiraishi

**Affiliations:** 1 Department of Electronic Science and Engineering, Kyoto University, Kyoto, 615-8510 Japan; 2 Institute for Chemical Research, Kyoto University, Uji, 611-0011 Japan; 3 Center for Spintronics Research Network, Kyoto University, Uji, 611-0011 Japan; 4 PRESTO, Japan Science and Technology Agency, Saitama, 332-0012 Japan

**Keywords:** Materials science, Physics

## Abstract

We demonstrate a robust method to dynamically tune magnon dissipation in yttrium iron garnet spheres by equipping a metallic band around the sphere’s equator, enabling precise control over magnon-photon coupling states. The collective magnetization dynamics in the YIG sphere induce circular eddy currents in the metallic band, whose magnitude can be systematically varied by adjusting the angle between the metallic band plane and an external static magnetic field. This angular dependence yields a pronounced modulation of the ferromagnetic resonance (FMR) linewidth, facilitating seamless transitions between the Purcell and strong coupling regimes without altering photon resonator parameters. Systematic FMR and resonator spectroscopy measurements confirm that eddy-current-induced losses govern the primary mechanism behind the observed tunable damping. By achieving extensive cos^2^*θ*‐type tunability of magnon relaxation rate, we precisely control the magnon-photon coupling state, approaching the critical coupling condition (*g* = *γ/2*). These results establish the YIG-metallic-band platform as a versatile and practical approach for engineering tunable magnon-polariton systems and advancing magnonic applications, including those exploring non-Hermitian magnonics.

**Supplementary Information:**

The online version contains supplementary material available at 10.1038/s41598-026-55914-x.

Magnons—quantized spin waves—have recently emerged as one of the central physical objects in condensed matter physics, offering a rich platform for both fundamental physics exploration and practical applications. Over the past few decades, magnons have attracted considerable attention for constructing quantum hybrid systems, owing to their robust capacity for energy exchange with other quantum information media, including photons^1–3^, phonons^4,5^, and additional magnon modes^6,7^. Of particular interest is the strong magnon-photon coupling that gives rise to magnon-polaritons, fueling advances in quantum memory^8–10^ and quantum transduction^11^. A crucial milestone in the study of magnon polaritons has been achieving controllable transitions between weak and strong coupling regimes, accomplished through temperature-dependent magnetization^12^ and precise tuning of the coupling via photon mode^13^. These developments have further enabled exploration into novel phenomena represented by non-Hermitian magnon-photon polariton systems^14–20^.

Central to much of this progress is the ferrimagnetic insulator yttrium iron garnet (Y_3_Fe_5_O_12_​, YIG). Spherical YIG, characterized by ultralow damping and bulk ferromagnetic properties, has been instrumental in advancing strong magnon-photon coupling research^3,9,21–26^, significantly enriching our understanding of magnon-photon interactions. However, while the large magnetic volume of millimeter-scale YIG spheres is advantageous for achieving strong coupling, it also predominantly ties the magnon mode to intrinsic magnetization states governed by the external magnetic field. This dependence complicates the incorporation of additional control mechanisms, thereby limiting the versatility of these systems in tunable magnon-polariton applications. Temperature-based tuning of magnetization^12^, although expected as a potential mechanism, inadvertently modifies the parameters of coupled photon modes, thereby complicating device integration and precise system coordination. Alternatively, surface-sensitive control methods—such as spin pumping, modulation of interfacial magnetic anisotropy^27–32^, or spin-torque applications via the spin Hall effect^33,34^—offer finer spatial resolution but remain fundamentally incompatible with large-scale YIG resonators due to their limited effective range on the nanoscale. Thus, the lack of a broadly applicable, scalable approach for tuning magnons in large YIG spheres remains a significant obstacle hindering further advancements in magnon-polariton-based technologies.

To overcome the challenge of robust magnon control in large-scale YIG spheres, we introduced a metallic band structure encircling the equator of the sphere, as illustrated in Fig. 1 Eddy currents induced within this metallic band by collective magnetization dynamics are expected to strongly couple to the extensive magnon volume present in the YIG sphere^35,36^, thus providing a promising mechanism for effective magnon manipulation. By systematically rotating the YIG sphere to adjust the orientation of the metallic band relative to the external static magnetic field, we achieved precise modulation of the dissipation rate of uniform magnons. This dissipation exhibited a distinct angular dependence, clearly arising from eddy currents generated by uniform magnons within the metallic structure rather than conventional surface-based phenomena such as spin pumping or interfacial effects. Crucially, our approach maintains photon mode parameters unchanged, enabling dynamic transitions between the Purcell regime and the strong-coupling regime in magnon-polariton systems. Our finding significantly advances the integration potential of YIG spheres within sophisticated magnonic platforms, particularly those exploring the physics of non-Hermitian magnon-polariton systems.

**Fig. 1:**
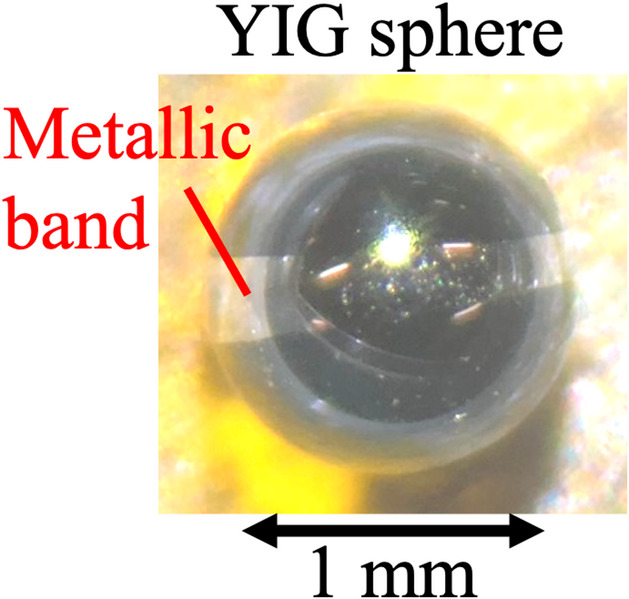
Image of YIG sphere with a metallic band.

To investigate the effects of adjacent metallic bands on magnon damping, we prepared three types of YIG spheres: a bare YIG sphere (sample-bare), a YIG sphere with a SiO₂/Cu/SiO₂ band (sample-SiO₂/Cu), and a YIG sphere with a Pt band (sample-Pt). Sample-SiO₂/Cu was designed to assess the impact of the metallic band as a conductor on magnon modes while eliminating spintronic effects such as spin pumping or the inverse spin Hall effect. This was achieved by using Cu, a material with weak spin–orbit interaction, and preventing direct contact between the metallic band and the YIG sphere through an insulating SiO₂ layer. Sample-Pt, in contrast, was used in photon-magnon coupling experiments because the SiO₂/Cu structure is mechanically fragile, and Cu degrades under prolonged atmospheric exposure.

Commercially available single crystalline YIG spheres, having a uniform diameter of 1 mm, were used in this study. All YIG spheres had a uniform diameter of 1 mm. To prepare a metallic band on each YIG sphere, a YIG sphere was immersed in PMMA (polymethyl methacrylate) resist to a depth of approximately 0.4 mm, and the resist on the surface was allowed to dry in air. The same procedure was repeated on the opposite side of the YIG sphere. This two-step dip-coating process left an uncoated equatorial zone while covering the remaining surface with the resist. Each resist-patterned sphere was mounted resist-side-down on the stage of a sputtering system. The base pressures immediately before sputtering were 3.4 × 10^−5^ Pa for Pt and 2.5 × 10^−5^ Pa for the SiO₂/Cu/SiO₂ trilayer. Argon was introduced at 0.5 Pa to sustain the plasma for all sputtering. Sputtering conditions were shown in Table 1. After deposition, lift-off of the resist yielded metal bands confined to the central region of each sphere; the band width was approximately 0.2 mm in both cases.

**Table 1:** Sputtering conditions of the thin film bands on the YIG sphere samples.

	Sputtered material	Deposition rate [nm/min]	Deposition time [min]
Sample-Pt	Pt	2.5	30
Sample-SiO_2_/Cu	SiO_2_ layer adjacent to YIG	2.3	15
	Cu layer	6.4	10
	SiO_2_ capping layer	2.3	10

The influence of the metallic bands on magnons excited within the YIG spheres was studied by measuring the absorption spectra of the samples placed on a coplanar waveguide (CPW), as shown in Fig. 2a. The YIG spheres were mounted on the center conductor of the CPW, with the metallic bands oriented parallel to the waveguide plane. A static magnetic field *H* was applied either in-plane (IP) or out-of-plane (OOP) with respect to the CPW. A 0 dBm microwave signal, sweeping frequencies from 0.02 to 9 GHz in 0.02 GHz increments, was supplied via a vector network analyzer (VNA) to generate an AC magnetic field on the CPW. The transmission parameter *S*_21_​ was recorded while sweeping the static magnetic field.

**Fig. 2:**
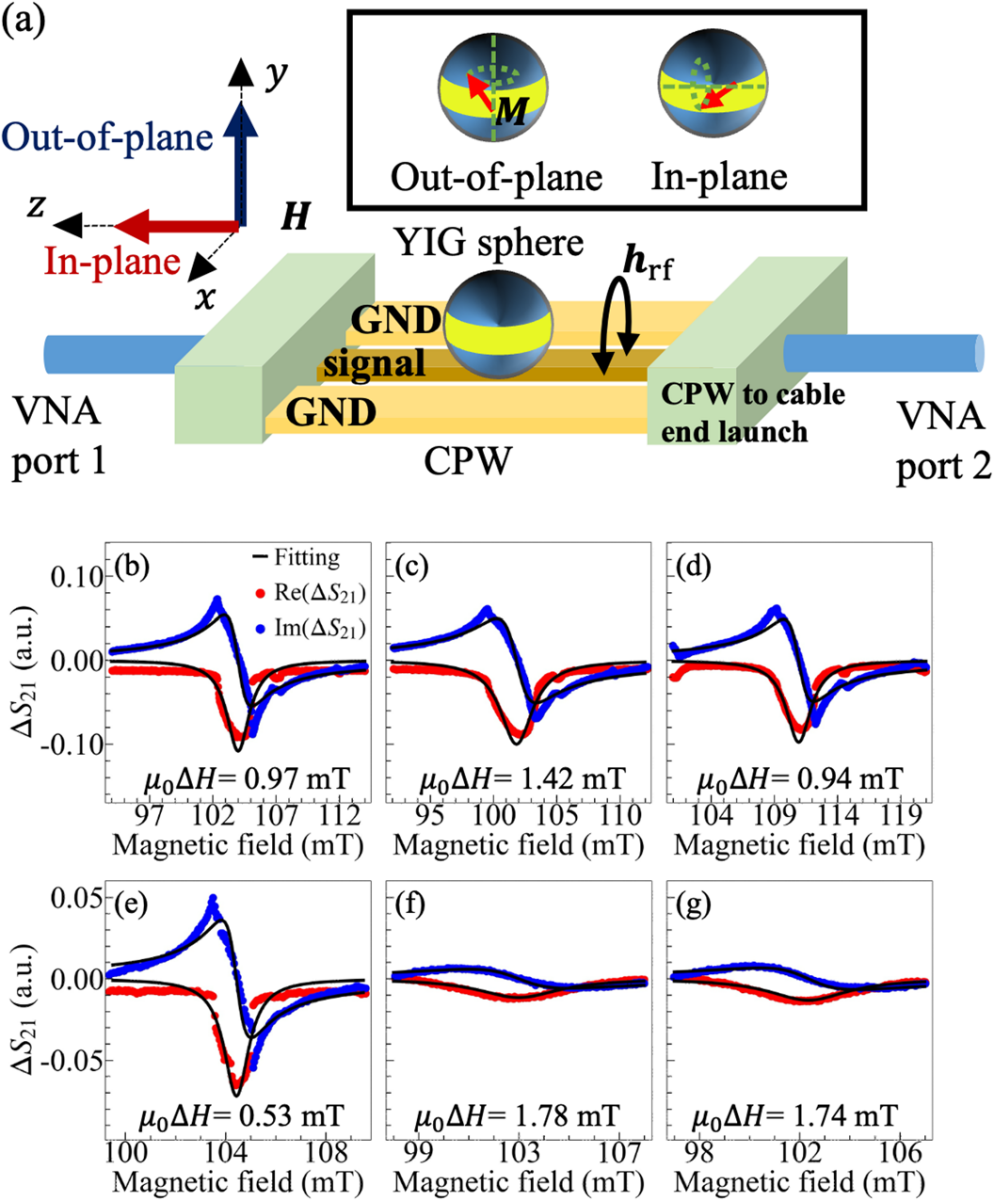
(**a**) Schematic measurement setup for CPW-FMR with the coordinate axes of the experimental system. The CPW was connected to ports of the VNA. Samples were placed on the signal line of the CPW. The inset image shows the relation between the metallic band plane and magnetization direction, where in-plane and out-of-plane direction align with the z and y axis, respectively. (**b**–**g**) Real parts (red circles) and imaginary parts (blue circles) of $\Delta {S}_{21}$ at 2.6 GHz in (**b**–**d**) OOP magnetic field configuration and (**e**–**g**) IP magnetic field configuration, where solid lines are fitting lines expressed by Eq. (1) and the estimated ${\mu}_{0}\Delta H$ are shown. The measured samples are (**b**, **e**) sample-bare, (**c**, **f**) sample-SiO_2_/Cu, and (**d**, **g**) sample-Pt, respectively.

The relationship between the susceptibility (*χ*) measured in the CPW-VNA setup and the normalized deviation ($\Delta {S}_{21}$) is given by $\Delta {S}_{21}=-\mathrm{j}A\omega \chi$, where *A* and *ω* are the amplitude constant and angular frequency of the AC magnetic field, respectively. For a ferromagnetic sphere with a metallic band, the susceptibility (*χ*) can be expressed as:

1 $$\chi =\frac{{\mu}_{0}M({\mu}_{0}H+\mathrm{j}{\mu}_{0}\Delta H)}{{({\mu}_{0}H+\mathrm{j}{\mu}_{0}\Delta H)}^{2}-{\omega }^{2}/{\gamma }^{2}},$$

where ${\mu}_{0}$ is the vacuum permeability, ${\mu}_{0}M$ the saturation magnetization, ${\mu}_{0}H$ the static magnetic field, ${\mu}_{0}\Delta H$ the half-width at half-maximum (HWHM) of the FMR spectra, and *γ* the gyromagnetic ratio. Figure 2b–g shows the extracted FMR spectra from the CPW-VNA measurements at a 2.6 GHz microwave frequency.

While the FMR spectra of all samples exhibited similar shapes in the OOP configuration, sample-SiO₂/Cu and -Pt displayed significantly broader linewidths in the IP configuration compared to the OOP configuration. The ${\mu}_{0}\Delta H$ values obtained by fitting Eq. (1) for sample-bare, sample-SiO₂/Cu, and sample-Pt in the OOP configuration are 0.97 ± 0.32 mT, 1.42 ± 0.34 mT, and 0.94 ± 0.42 mT, respectively. However, in the IP configuration, ${\mu}_{0}\Delta H$ is enhanced to 1.78 ± 0.14 mT for sample-SiO₂/Cu and 1.74 ± 0.13 mT for sample-Pt, whereas no enhancement is observed for sample-bare. This comparable enhancement in ${\mu}_{0}\Delta H$ for the two metallic band samples suggests that interfacial damping effects, such as spin pumping due to the Pt band, are not the primary contributors. Instead, the observed damping enhancement in the IP configuration is likely due to eddy currents induced in the metallic bands by the electromagnetic induction effect arising from the magnetization dynamics of the YIG sphere.

To quantitatively analyze the enhancement of magnon damping induced by the metallic bands in the in-plane configuration, we evaluate the susceptibility using the coordinate system defined in Fig. 2a. In the in-plane configuration, i) the dipolar field generated by the ac component of the magnetization creates sinusoidal interlinked magnetic flux in the area circled by the metallic band. The resultant circular ac charge current generates an Ampère magnetic field. Additionally, ii) a possible contribution from the electric current driven by the inverse spin Hall effect (iSHE) due to the ac spin current pumped from the precessing magnetization in YIG is included. The spin-current amplitude vector is proportional to the outer product of magnetization and its time derivative^37^. These two effects are neglected in the out-of-plane configuration, where only a longitudinal charge current along the metal band with an ideal zero-width band is considered. Consequently, the effective magnetic-field components in the in-plane configuration are given by:

2a $${H}_{x}={h}_{\mathrm{r}\mathrm{f}}{e}^{\mathrm{j}\omega t}-{N}_{x}{M}_{x}{e}^{\mathrm{j}\omega t},$$

2b $${H}_{y}=-{N}_{y}{M}_{y}{e}^{\mathrm{j}\omega t}-\mathrm{j}\omega \beta {M}_{y}{e}^{\mathrm{j}\omega t}+\omega \varepsilon {M}_{y}{e}^{\mathrm{j}\omega t},$$

2c $${H}_{z}=H-{N}_{z}{M}_{z},$$

where, ${H}_{i}, {M}_{i}, {N}_{i}$ denote magnetic field, magnetization and demagnetizing factor along the direction *i*-axis (*i* = *x*, *y*, and z), respectively. Here, *H* is dc magnetic field, while *β* and *\varepsilon* represent the coefficients associated with the eddy-current and iSHE-induced electric-current contributions, respectively. The coefficients *β* and *\varepsilon* have dimensions of the reciprocal of angular frequency in the unit of s/rad. In the present coordinate system, ${h}_{\mathrm{r}\mathrm{f}}$ denotes the dominant x-component of the rf magnetic field generated by the CPW; the possible y-component arising from field inhomogeneity is assumed to be negligible, as discussed in detail in Supplemental Material G. Substituting Eqs. (2) into the Landau-Lifshitz-Gilbert (LLG) equation,

3 $$\frac{{\mathrm d}{\boldsymbol{M}}}{{\mathrm d}t}=-\gamma {\boldsymbol{M}}\times {\boldsymbol{H}}+\frac{\alpha }{{M}_{\mathrm{s}}}{\boldsymbol{M}}\times \frac{{\mathrm d}{\boldsymbol{M}}}{{\mathrm d}t},$$

the component equations of magnetization become,

4a $$\mathrm{j}\omega {M}_{x}=-\gamma \left[H+\mathrm{j}\omega {\beta M}_{z}-\omega \varepsilon {M}_{z}+\frac{\mathrm{j}\omega \alpha }{\gamma }\right]{M}_{y},$$

4b $$\mathrm{j}\omega {M}_{y}=-\gamma \left[{M}_{z}{h}_{\mathrm{r}\mathrm{f}}-{M}_{x}\left({H}_{\mathrm{D}\mathrm{C}}+\frac{\mathrm{j}\omega \alpha }{\gamma }\right)\right].$$

Solving these coupled equations gives the susceptibility *χ*,

5 $$\chi =\frac{{\mu}_{0}{M}_{x}}{{\mu}_{0}{h}_{\mathrm{r}\mathrm{f}}}=\frac{{{\mu}_{0}M}_{z} \left[{\mu}_{0}H+\frac{\mathrm{j}\omega \alpha }{\gamma }+\mathrm{j}\omega \beta {{\mu}_{0}M}_{z}-\omega \varepsilon {\mu}_{0}{M}_{z}\right]}{\left[{\mu}_{0}H+\frac{\mathrm{j}\omega \alpha }{\gamma }\right]\left[{\mu}_{0}H+\frac{\mathrm{j}\omega \alpha }{\gamma }+\mathrm{j}\omega \beta {\mu}_{0}{M}_{z}-\omega \varepsilon {{\mu}_{0}M}_{z}\right]-\frac{{\omega }^{2}}{{\gamma }^{2}}}.$$

Equation (5) allows us to fit the FMR spectra to extract the coefficients *β* and *\varepsilon*, corresponding to the eddy-current and iSHE contributions, respectively. The values of *β* for sample-Cu/SiO_2_ and -Pt are estimated to be 0.80 × 10^–12^ s/rad and 0.96 × 10^–12^ s/rad, respectively, as shown in Fig. 3a and b, whereas the iSHE contribution is negligibly small compared with *β*.

**Fig. 3:**
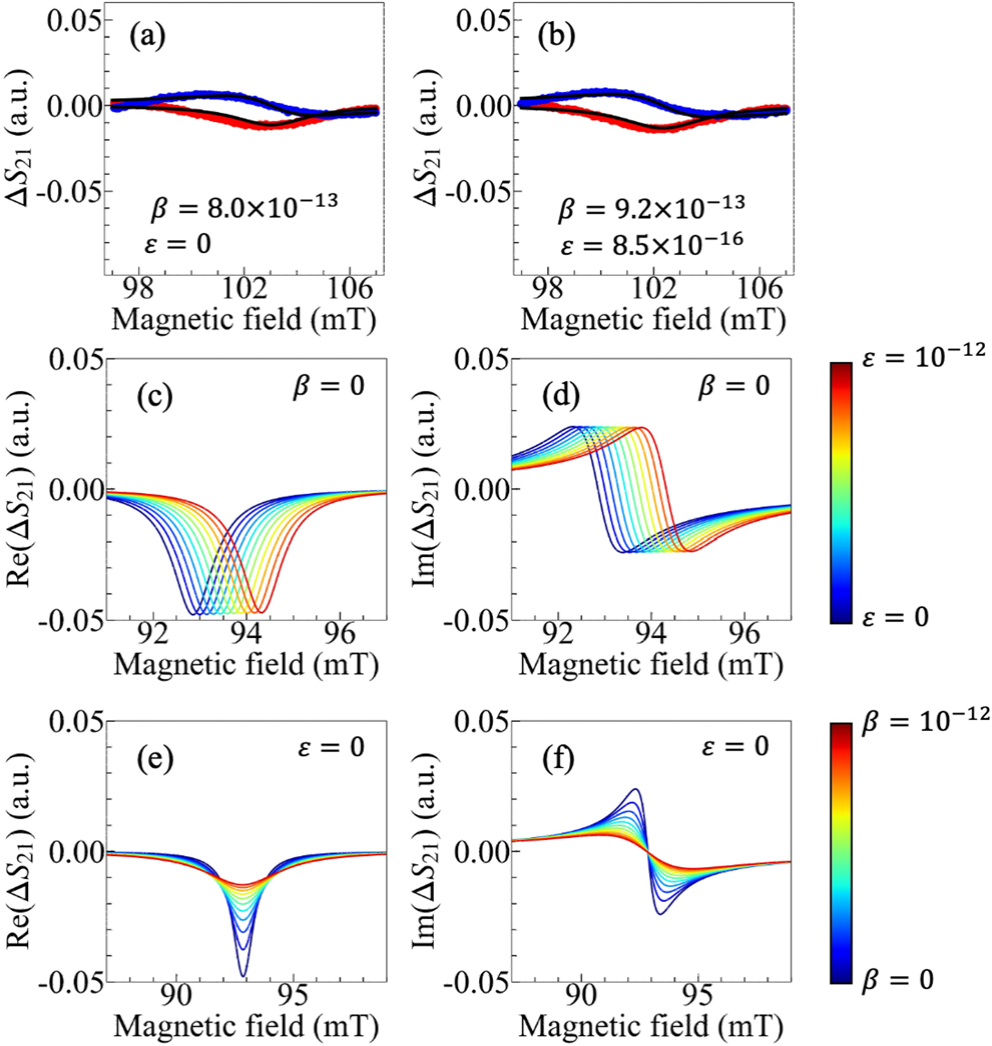
(**a**, **b**) Measured *ΔS*_21_ and its fittings (solid lines) of real parts (red circles) and imaginary parts (blue parts) of *S*_21_ at 2.6 GHz using Eq. (5). The measured samples are (**a**) Sample-SiO_2_/Cu and (**b**) Sample-Pt, respectively. (**c**, **d**) The simulated transitions of (**c**) real parts and (**d**) imaginary parts of *S*_21_ when varying *\varepsilon* from 0 to 10^–12^ with *β* = 0. The resonance field shifts as *\varepsilon* increases while the linewidth does not change significantly. (**e**, **f**) The simulated transitions of (**e**) real parts and (**f**) imaginary parts of *S*_21_ with varying *β* from 0 to 10^–12^ with *\varepsilon* = 0. The linewidth increases as *β* is increased.

Figure 3c–f show the transitions of *S*_21_ as *\varepsilon* varies from 0 to 10^–12^ s/rad with *β =* 0 s/rad [(c) and (d)] and as *β* varies from 0 to 10^–12^ s/rad with *\varepsilon =* 0 s/rad [(e) and (f)] in Eq. (5). These results indicate that the eddy current increases the linewidth, while the electric current via iSHE shifts the resonant field of FMR. The results in Fig. 3a and b clearly show that the attached metallic band modulates the resonance linewidth rather than the resonance field, in agreement with the eddy-current mechanism and incompatible with the iSHE contribution. These findings strongly indicate that the modulation of magnon relaxation in metallic band-adjacent YIG spheres originates from the circular eddy currents excited by the magnetization dynamics.

Motivated by the above analysis, we next examine the angular dependence of the FMR linewidth, expecting that the magnon damping in the YIG sphere can be finely tuned according to the angle between the external magnetic field and the metallic band plane. The magnetic angular dependence of ${\mu}_{0}\Delta H$ was experimentally determined by using ESR cavity enabling to measure the FMR linewidth as the angle of the external magnetic field was rotated in a plane perpendicular to the metallic band, as illustrated in Fig. 4a. In this measurement setup, the uniform TE_011_ microwave photon mode with the quality factor around 9000 strongly excites the Kittel mode of the YIG sphere. The samples were placed in a rectangular resonator, with the static magnetic field swept along the—y direction. The microwave frequency and power were fixed at 9.12 GHz and 0 dBm, respectively. The rotation angle (*θ*) for samples with metallic bands was defined as the angle between the metallic band and the static magnetic field.

**Fig. 4:**
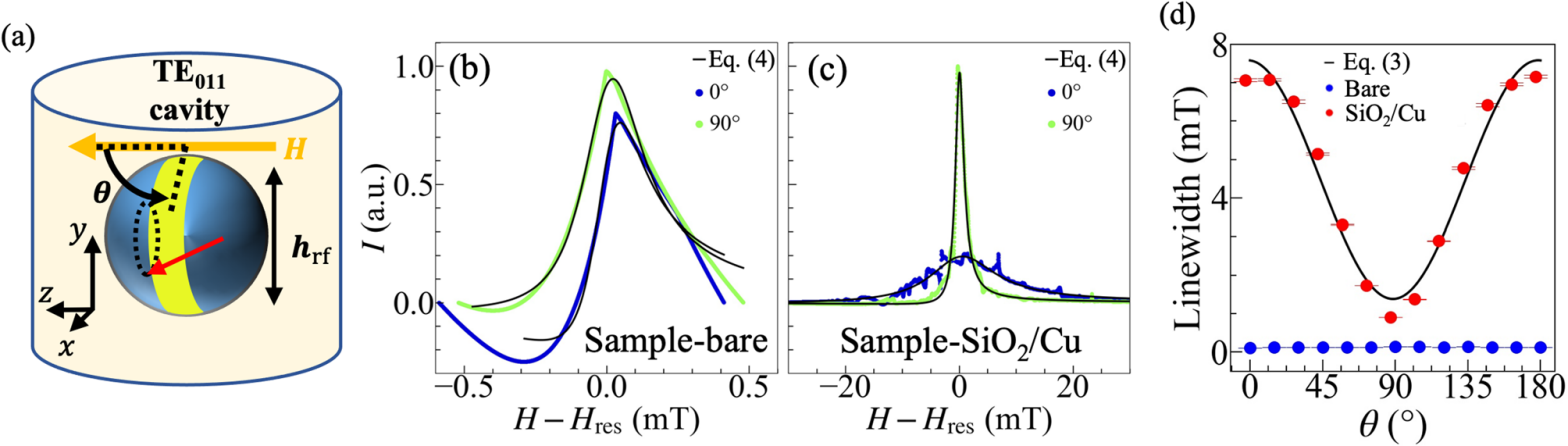
(**a**) Schematic image of FMR measurement using ESR cavity (ESR-FMR), where *θ* is the angle between the external magnetic field and the metallic band plane. *θ* = 0° and 90° correspond with the in-plane and out-of-plane configurations, respectively. YIG spherical samples are mounted on the tip of the quartz rod. (**b**, **c**) FMR spectra of *θ* = 0° and 90° for sample-bare and -SiO_2_/Cu, respectively. (**d**) *θ*-dependence of the estimated ${\mu}_{0}\Delta H$ for sample-bare and -SiO_2_/Cu.

Figure 4b and c depict the FMR spectra for sample-bare and sample-SiO₂/Cu at *θ* = 0° and 90°. While the FMR spectra of sample-bare remain largely unchanged, the linewidth of the spectra for sample-SiO₂/Cu is significantly modulated as *θ* changes. The spectra can be described using the following equation^31^:

6 $$I=\frac{S{({\mu}_{0}\Delta H)}^{2}}{{({\mu}_{0}H-{\mu}_{0}{H}_{\mathrm{r}\mathrm{e}\mathrm{s}})}^{2}+{({\mu}_{0}\Delta H)}^{2}}+\frac{A\left({\mu}_{0}H-{\mu}_{0}{H}_{\mathrm{r}\mathrm{e}\mathrm{s}}\right){\mu}_{0}\Delta H}{{({\mu}_{0}H-{\mu}_{0}{H}_{\mathrm{r}\mathrm{e}\mathrm{s}})}^{2}+{({\mu}_{0}\Delta H)}^{2}} ,$$

where *I*, ${\mu}_{0}H$, ${\mu}_{0}{H}_{\mathrm{r}\mathrm{e}\mathrm{s}}$, ${\mu}_{0}\Delta H$, *S*, and *A* are intensity of spectra, the static magnetic field, the resonance field, the HWHM of FMR, symmetric and asymmetric components, respectively. For sample-bare, the estimated ${\mu}_{0}\Delta H$ values are 1.05 ± 0.00 mT (*θ* = 0°) and 1.32 ± 0.00 mT (*θ* = 90°). In contrast, sample-SiO₂/Cu shows significantly different values: 7.07 ± 0.04 mT (*θ* = 0°) and 0.91 ± 0.04 mT (*θ* = 90°). The enhanced damping for the *k* = 0 magnon mode, where the precession angle is parallel to the band plane, aligns with the experimental results obtained using the CPW. This finding highlights the key role of angular dependence in determining the dissipation properties of magnons in the presence of metallic bands. Both sample-SiO_2_/Cu and -Pt exhibit comparable angular dependences of the magnon damping in the CPW-FMR measurement. In sample-SiO_2_/Cu, the insulating SiO_2_ layer prevents direct electrical contact between the YIG and the Cu, thereby eliminating spin pumping and spin–orbit torque effects, which require direct ferromagnet-nonmagnet contact. If these interfacial effects were dominant, the angular dependence of the magnon damping shown in Figs. 2 and 4 would vanish in sample-SiO_2_/Cu. The results obtained from the samples with SiO_2_/Cu and with Pt thus indicates that the spintronic effects are negligible in both cases, and the finely tunable angle-dependent magnon damping induced by the metallic band is attributed to the eddy-current contribution.

Figure 4d presents the angular dependence of the estimated ${\mu}_{0}\Delta H$ for both sample-bare and sample-SiO_2_​/Cu using Eq. (6) as the fitting function of the FMR spectra. Notably, the magnon damping in the YIG sphere with Cu/SiO_2_​ is strongly modulated by varying the angle of the external magnetic field, whereas the FMR linewidth of sample-bare remains constant. Based on the CPW-FMR results, this pronounced modulation can be attributed to eddy-current loss in the metallic band. To clarify the mechanism underlying the modulation of magnon dissipation modulation, we examine the FMR linewidth as a function of the magnetic field orientation from both experimental and theoretical perspectives.

For the case of ESR-FMR experiments, we analyze the susceptibility, in a manner analogous to the VNA experiments, for taking into account the angle between the magnetic field and the plane of the metallic band. The components of the magnetic fields are then given by:

7a $${H}_{x}=-{N}_{x}{M}_{x}{e}^{\mathrm{j}\omega t}-\mathrm{j}\omega {M}_{x}\beta {\mathrm{c}\mathrm{o}\mathrm{s}}^{2}\theta {e}^{\mathrm{j}\omega t},$$

7b $${H}_{y}={h}_{\mathrm{r}\mathrm{f}}{e}^{\mathrm{j}\omega t}-{N}_{y}{M}_{y}{e}^{\mathrm{j}\omega t},$$

7c $${H}_{z}=H-{N}_{z}{M}_{z}-\mathrm{j}\omega {M}_{x}\beta \mathrm{c}\mathrm{o}\mathrm{s}\theta \mathrm{s}\mathrm{i}\mathrm{n}\theta {e}^{\mathrm{j}\omega t},$$

where the coordinate system is chosen consistently with CPW-FMR and ESR-FMR geometries shown in Figs. 2a and 4a. Note that the coefficient for the iSHE-induced current *\varepsilon*, which causes a resonance-field shift as discussed above, is neglected here since sample-SiO_2_/Cu was used for the angle-dependent ESR-FMR measurements. By substituting Eqs. (7) into the LLG equation and neglecting high order terms, we obtain the following expression for the susceptibility,

8 $$\chi =\frac{{\mu}_{0}{M}_{y}}{{\mu}_{0}{h}_{\mathrm{r}\mathrm{f}}}=\frac{{\mu}_{0}{M}_{z}\left[{\mu}_{0}H+\frac{\mathrm{j}\omega \alpha }{\gamma }+\mathrm{j}\omega \beta {{\mu}_{0}M}_{z}{\mathrm{c}\mathrm{o}\mathrm{s}}^{2}\theta \right]}{\left[{\mu}_{0}H+\frac{\mathrm{j}\omega \alpha }{\gamma }\right]\left[{\mu}_{0}H+\frac{\mathrm{j}\omega \alpha }{\gamma }+\mathrm{j}\omega \beta {\mu}_{0}{M}_{z}{\mathrm{c}\mathrm{o}\mathrm{s}}^{2}\theta \right]-\frac{{\omega }^{2}}{{\gamma }^{2}}}.$$

Here, we replace $\frac{\omega }{\gamma }$ as ${H}^{\prime}$ and $H-{H}^{\prime}=\Delta H$. Under the conditions ${\alpha }^{2}\ll 1, \alpha \beta \gamma {\mu}_{0}{M}_{z}\ll 1$, and $\Delta H\ll H$, Eq. (8) can be rewritten as

9 $$\chi =\frac{{\mu}_{0}{M}_{z}}{2{\mu}_{0}{H}^{\prime}}\frac{\left[{\mu}_{0}H+\mathrm{j}\left(\frac{\omega \alpha }{\gamma }+\omega \beta {\mu}_{0}{M}_{z}{\mathrm{c}\mathrm{o}\mathrm{s}}^{2}\theta \right)\right]\left[{\mu}_{0}\Delta H-\mathrm{j}\left(\frac{\omega \alpha }{\gamma }+\frac{\omega \beta {\mu}_{0}{M}_{z}{\mathrm{c}\mathrm{o}\mathrm{s}}^{2}\theta }{2}\right)\left(1+\frac{\Delta H}{{H}^{\prime}}\right)\right]}{{\left({\mu}_{0}\Delta H\right)}^{2}+{\left(\frac{\omega \alpha }{\gamma }+\frac{\omega \beta {\mu}_{0}{M}_{z}{\mathrm{c}\mathrm{o}\mathrm{s}}^{2}\theta }{2}\right)}^{2}\left(1+\frac{2\Delta H}{{H}^{\prime}}\right)}.$$

The intensity of FMR spectra is proportional to imaginary part of *χ*, which becomes

10 $$\mathrm{I}\mathrm{m}\left(\chi \right)=-\frac{{\mu}_{0}{M}_{z}}{2{\mu}_{0}{H}^{\prime}}\frac{\frac{\omega \alpha }{\gamma }\left({\mu}_{0}H-{\mu}_{0}{H}^{\prime}\right)+{\mu}_{0}{H}^{\prime}C}{{\left[{\mu}_{0}H-\left({\mu}_{0}{H}^{\prime}-\frac{{C}^{2}}{{\mu}_{0}{H}^{\prime}}\right)\right]}^{2}+{C}^{2}-\frac{{C}^{4}}{{\left({\mu}_{0}{H}^{\prime}\right)}^{2}}},$$

where, $C=\frac{\omega \alpha }{\gamma }+\frac{\omega \beta {\mu}_{0}{M}_{z}{\mathrm{c}\mathrm{o}\mathrm{s}}^{2}\theta }{2}$. For $C\ll {\mu}_{0}{H}^{\prime}$, we have ${C}^{2}-\frac{{C}^{4}}{{\left({\mu}_{0}{H}^{\prime}\right)}^{2}}={C}^{2}\left[1-{\left(\frac{C}{{\mu}_{0}{H}^{\prime}}\right)}^{2}\right]\approx {C}^{2}$. Under this approximation, Eq. (10) reduces to the same Lorentzian as Eq. (5) and FMR linewidth of spectra is given by

11 $${\mu}_{0}\Delta H= \frac{\omega \alpha }{\gamma }+\frac{\omega \beta {\mu}_{0}{M}_{z}{\mathrm{c}\mathrm{o}\mathrm{s}}^{2}\theta }{2}.$$

Since the coefficient of the angle-dependent linewidth is directly proportional to the eddy-current-induced coefficient *β*, the experimental results on angle-dependent magnon damping allow us to estimate the value of *β*. The extracted parameter *β* for sample-SiO_2_​/Cu is 1.22 × 10^–12^ s/rad, which is comparable to the value obtained from the CPW-FMR measurement through fitting the spectra shown in Fig. 3a with Eq. (5).

Invoking Ampère’s law for the inductive circular current excited in the metallic band by uniform magnetization dynamics, one approximately obtains the relation ${\beta}_{\mathrm{D}\mathrm{C}}=\frac{\uppi r{\mu}_{0}}{2R}$, where *r* and *R* denote the radius and resistance of the metallic band, respectively. A typical two-terminal *I*-*V* measurement of the metallic band yields *R* as 356 Ω (see Supplemental Materials), leading to *β =* 2.77 × 10^–12^ s/rad. The agreement of ${\beta}_{\mathrm{D}\mathrm{C}}$ within the same order of magnitude of *β* estimated from independent measurements provides strong evidence that the circular ac current in the metallic band effectively suppresses magnons in the adjacent YIG sphere. More than a factor of two differences may stem from the difference of estimation method for *β* between DC and RF estimation. In practice, additional factors—such as contact geometry and possible frequency-dependent conductivity—can contribute to difference between DC and high-frequency behavior. Building on the above discussions, rotating the magnetic field relative to the plane of the metallic band modulates the interaction between the eddy-current-induced circular current and the magnetization dynamics in the YIG sphere, thus offering a promising route for the fine-tuning of magnon damping.

The magnon–photon coupling state is characterized by the coupling strength *g*, the magnon relaxation rate ${\kappa}_{\mathrm{m}}$, and the photon relaxation rate ${\kappa}_{\mathrm{p}}$ *[2]*. Because ${\kappa}_{\mathrm{m}}$ in a YIG sphere with a metallic band can be controlled by changing the angle of the external magnetic field, as demonstrated in the experiments above, such a YIG sphere serves as a key component for achieving tunable strong magnon–photon coupling. In particular, the circular eddy current in the metallic band adjacent to the YIG sphere—rather than spintronic spin-to-charge conversion—can significantly affect the magnon dynamics as we explored. Therefore, we employed sample-Pt to demonstrate finely tunable magnon–photon coupling.

A TiO_2_ (rutile) cylindrical resonator^38^ was used to mitigate photon losses and strongly confine photon mode for the achievement of strong magnon-photon coupling state. As shown in Fig. 5a, the sample–Pt assembly, attached to a rotatable rod, was inserted into the rutile resonator with a designed resonance frequency of 2.61 GHz. The resonator was mounted on a sapphire substrate and placed in a Cu shield to suppress radiation losses. The microwave reflection *S*_11_​ was measured via a circular coil antenna adjacent to the rutile resonator, while a static magnetic field was applied by an electromagnet. The extracted *S*_11_ was divided the background reflection measured without rutile resonator (${S}_{11}^{\mathrm{B}\mathrm{G}}$) to remove the background noise from the measurement set-up. Here the quality factor of rutile resonator without YIG sphere is determined to be around 2270, which is estimated by fitting the transmission spectra measured by VNA. The angle *θ* between the external magnetic field and the plane of the metallic band was controlled by rotating the rod supporting the YIG sphere.

Figure 5c–e present the magnitude of |*S*_11_/${S}_{11}^{\mathrm{B}\mathrm{G}}$| as a function of frequency and applied magnetic field at *θ* = 0°, 45°, and 90°, respectively. The spectrum for sample-bare is shown in Fig. 5b as a reference, where a prominent anticrossing is observed, confirming strong magnon-photon coupling between the YIG sphere and the rutile resonator. Even after implementing the metallic band into YIG sphere, a splitting appears at a certain value of the magnet current, indicating a strong interaction between the magnon mode in the YIG sphere and the photon mode in the rutile resonator. The variation in the resonant magnetic field values stem from the shift in the magnetic resonance field due to the crystalline magnetic anisotropy of YIG (see Supplemental Materials). Notably, the pronounced anti-crossing observed at *θ* = 90° contrasts with the nearly absent anti-crossing at *θ* = 0°. This behavior is consistent with the experimental observation that magnon damping is significantly enhanced when the external magnetic field is applied parallel to the band plane.

**Fig. 5:**
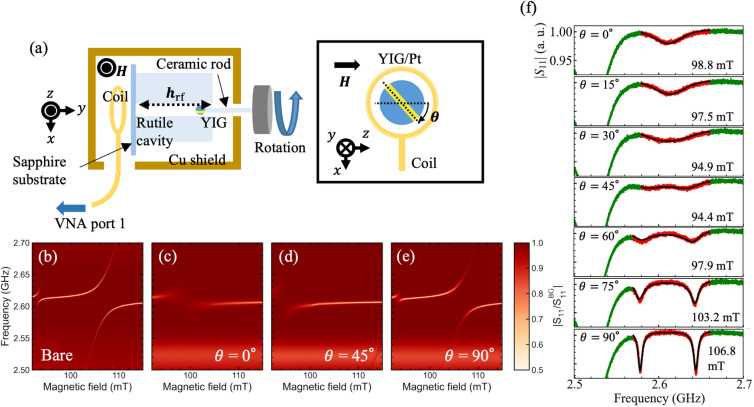
(**a**) Experimental setup for manipulation of magnon-polariton in YIG sphere. The rutile dielectric resonator was placed in a Cu shield to prevent the radiation loss of microwave. Sample-Pt attached with the ceramic rod was inserted into the rutile resonator, and spectra were measured with VNA while rotating sample-Pt by the rod. The rotation angle *θ* is shown in the inset. (**b**–**d**) Two-dimensional colormaps of |*S*_11_/${S}_{11}^{\mathrm{B}\mathrm{G}}$| for sample-Pt and sample-bare as a function of external magnetic field and frequency. Here, the spectrum for sample-bare is shown as the reference in (**b**) and the spectra for sample-Pt at different magnetic field angles (**c**) *θ* = 0°, (**d**) *θ* = 45° and (**e**) *θ* = 90°. (**b**), a prominent anti-crossing is observed (strong coupling) at (**b**) sample-bare and (**e**) *θ* = 90°. (**d**) shows the process of that transition. (**f**) |*S*_11_/${S}_{11}^{\mathrm{B}\mathrm{G}}$| at the magnetic field value at the resonant condition for each angle. The red data and black lines represent the fitting range and lines, respectively.

Figure 5f shows the *θ*-dependence of |*S*_11_/${S}_{11}^{\mathrm{B}\mathrm{G}}$| around the magnon resonance current. While only a single peak appears up to *θ* = 45°, the peak splits into two from *θ* = 45° to 90°. This transition signifies the onset of strong magnon–photon coupling as the external magnetic field orientation is tilted relative to the plane of the band.

To determine the coupling strength and relaxation rates of the magnon and photon modes, we employ the standard input–output theory^39^, which is a powerful way of estimating coupling parameters independently from frequency-dependent spectra. In this framework, the microwave reflection coefficient is given by

12 $${S}_{11}=\frac{i\left(\omega -{\omega}_{\mathrm{c}}\right)-\frac{{\kappa}_{\mathrm{i}}-{\kappa}_{\mathrm{e}}}{2}+\frac{{g}^{2}}{i\left(\omega -{\omega}_{\mathrm{m}}\right)-\frac{{\kappa}_{\mathrm{m}}}{2}}}{i\left(\omega -{\omega}_{\mathrm{c}}\right)-\frac{{\kappa}_{\mathrm{i}}+{\kappa}_{\mathrm{e}}}{2}+\frac{{g}^{2}}{i\left(\omega -{\omega}_{\mathrm{m}}\right)-\frac{{\kappa}_{\mathrm{m}}}{2}}},$$

where ${\omega}_{\mathrm{c}}$ and ${\omega}_{\mathrm{m}}$ are the resonance angular frequencies of the resonator and ferromagnetic resonance (FMR) mode, respectively, while ${\kappa}_{\mathrm{i}}$ and ${\kappa}_{\mathrm{e}}$​ represent the resonator’s internal dissipation and external coupling rates. Note that the fitting of the spectra is conducted at vacuum condition (${\omega}_{\mathrm{c}}={\omega}_{\mathrm{m}}$), which is a well-established way in previous works on strong magnon-photon coupling^3,25^. By fitting the experimental spectra at *θ* = 0°, 45° and 90° to Eq. (12), we obtain the system parameters summarized in Table 2.

**Table 2:** Estimated system parameters: *g/2π*, ${\kappa}_{\mathrm{m}}/4\pi$, ${\kappa}_{\mathrm{i}}$/4π obtained by fitting the data of |*S*_11_| at *θ* = 0°, 45°, 90°.

*θ*	*g/2π*(MHz)	${\kappa}_{\mathrm{m}}/4\pi$(MHz)	${\kappa}_{\mathrm{i}}/4\pi$(MHz)	Coupling state
0°	32.36 ± 0.55	67.05 ± 2.33	0.42 ± 0.01	Purcell (${\kappa}_{\mathrm{m}}/2> g>{\kappa}_{\mathrm{p}}/2)$
45°	34.58 ± 0.21	38.43 ± 0.51	0.32 ± 0.01	Compensating (${\kappa}_{\mathrm{m}}/2\sim g>{\kappa}_{\mathrm{p}}/2$)
90°	33.16 ± 0.03	5.80 ± 0.04	0.27 ± 0.01	Strong ($g> {\kappa}_{\mathrm{m}}/2,{\kappa}_{\mathrm{p}}/2$)

Based on these fitted parameters, the magnon–photon coupling states at different angles *θ* can be classified as follows: the Purcell regime (*θ* = 0°), near the compensating point ($g \sim {\kappa}_{\mathrm{m}}/2$) for *θ* = 45°, and the strong-coupling regime (*θ* = 90°). These assignments follow the criteria outlined in Ref.^2^ for distinguishing between different coupling states. Notably, the ability to realize these regimes simply by rotating the YIG sphere, which is surrounded by a metallic band, highlights the robust tunability of this system. Although the spectra in Fig. 5f exhibit a single peak from *θ* = 0° up to approximately *θ* = 45°, a progressive evolution into a split-peak structure emerges near *θ* = 90°. This gradual transition underscores the wide tuning range of the coupling conditions in this system. As discussed, this tunability stems from the angular dependence of the magnon relaxation rate γ, which is expected to follow a cos^2^*θ* -type behavior.

Figure 6a shows the *θ*-dependence of *g* and ${\kappa}_{\mathrm{m}}/2$, extracted from fittings to Eq. (12). Figure 6b and c show the angular dependence of ${\kappa}_{\mathrm{i}}$, ${\kappa}_{\mathrm{e}}$, and ${\kappa}_{\mathrm{p}}={\kappa}_{\mathrm{i}}+ {\kappa}_{\mathrm{e}}$ of YIG-Pt in the experiments described in the main text. The change of ${\kappa}_{\mathrm{i}}$ is artificial due to the alignment of Cu shield around rutile resonator while ${\kappa}_{\mathrm{e}}$ remains constant because the positions of the coil and the rutile resonator were fixed. Because the total resonator dissipation rate ${\kappa}_{\mathrm{p}}/2\pi =({\kappa}_{\mathrm{i}}+{\kappa}_{\mathrm{e}})/2\pi$ remains below 2 MHz at all angles, the coupling regime is classified as either the Purcell-effect regime (${\kappa}_{\mathrm{m}}/2>g>{\kappa}_{\mathrm{p}}/2$) or the strong-coupling regime ($g>{\kappa}_{\mathrm{p}}/2, {\kappa}_{\mathrm{m}}/2$). Interestingly, while *g* remains nearly constant with respect to *θ*, ${\kappa}_{\mathrm{m}}/2$ exhibits a clear cos^2^*θ* dependence, allowing the system to be tuned precisely to the condition $g= {\kappa}_{\mathrm{m}}/2$. This fine-tuning capacity is promising for constructing platforms dedicated to non-Hermitian physics.

**Fig. 6:**
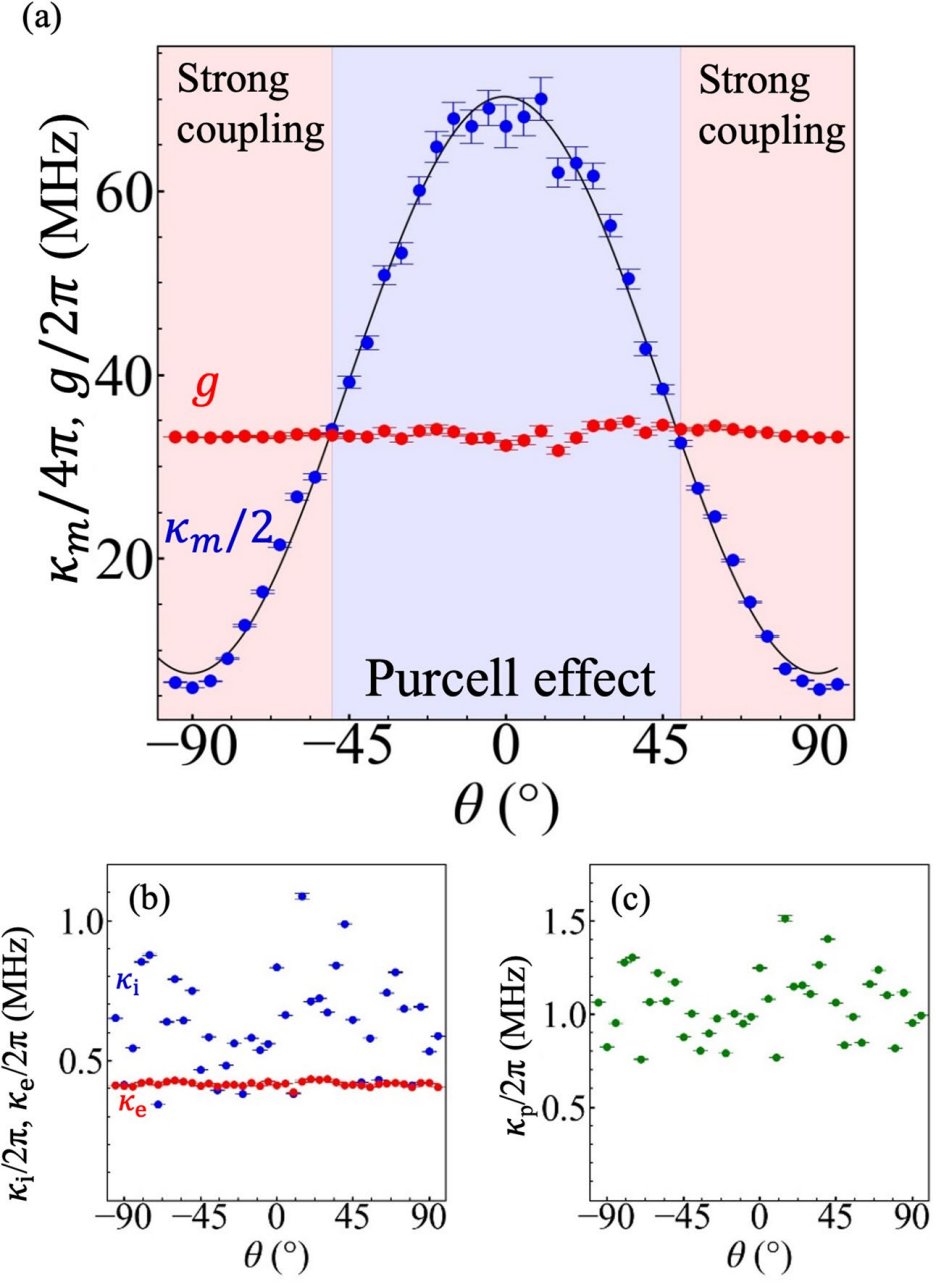
(**a**) *θ*-dependence of *g/2π* and ${\kappa}_{\mathrm{m}}/4\pi$ of magnon-polaritons in YIG sphere. The coupling state is considered to be in Purcell effect in the range approximately from − 50° to 50°, and strong coupling in the other range. The black line shows the fitting curve with the translated Eq. (4) in frequency range. (**b**) *θ*-dependence of ${\kappa}_{\mathrm{i}}$ (blue circles) and ${\kappa}_{\mathrm{e}}$ (red circles) of Sample-Pt. (**c**) *θ*-dependence dependence of ${\kappa}_{\mathrm{p}}$ of Sample-Pt.

Furthermore, ${\kappa}_{\mathrm{m}}$ can be related to the half-width at half-maximum (HWHM) in FMR through the dispersion relation *ω*/2π = *γμ*_0_|*H*|. We therefore apply the fitting procedure described in Eq. (11) to the ${\kappa}_{\mathrm{m}}$ data in Fig. 6a, yielding an excellent agreement with the experimental data and an extracted *β* = 1.63 ± 0.03 × 10^–12^ s/rad, comparable in the same order of magnitude with the results from ESR-FMR measurements. Note that we do not directly compare *β* from the CPW measurement with that from the resonator measurements, since the excited microwave field distribution in CPW is more inhomogeneous in mm-space than in the resonator, which can reasonably lead to systematic differences in the extracted *β*. These findings corroborate highly effective modification of the magnon dissipation in a YIG sphere by simply adjusting the orientation of the external static magnetic field relative to the metallic band. This technique achieves sufficient tunability to traverse multiple coupling regimes, thereby providing a valuable tool for realizing long-anticipated milestones in non-Hermitian magnonics.

Before concluding, we turn our attention to the design principles and possible future developments for this mechanism. In the present study, we employed a resist-patterned metallic thin-film band as a well-defined conductive path around the YIG sphere. Although a metallic wire loop could also induce a similar eddy-current effect in principle, its larger metallic volume may introduce additional microwave loss and thereby reduce the photon Q factor of the rutile resonator. The thin-film band geometry is therefore advantageous for tuning the magnon damping while preserving a sufficiently high photon Q factor, which is essential for coherent magnon-photon coupling. The demonstrated method of controlling magnon dissipation in a YIG sphere can also be viewed as modulating the coupling strength between the metallic band and the YIG sphere. Consequently, this opens the door to introducing tunable coupling strength with switchable transducer functionality in the ring resonator–YIG sphere system proposed in Refs.^23,40^. Moreover, since *β* is inversely proportional to the metallic band’s resistance, exploiting the ultralow resistance of a superconductor provides a route to increase *β* significantly. The combined system of superconductor and YIG sphere studied in Ref.^23^ broadens the modulation range and thus enables wide tunability of the magnon–polariton coupling state in the YIG sphere.

In conclusion, we have shown that by rotating the relative angle of the external magnetic field to a metallic band on a YIG sphere, the magnon–photon coupling state can be tuned between the Purcell and strong coupling regime. This approach modulates the magnon relaxation rate ${\kappa}_{\mathrm{m}}$ via eddy currents in the metallic band, yielding a cos^2^*θ*-type sinusoidal control of magnon damping while maintaining a constant coupling strength. Such tunability, combined with the inherently low damping of YIG, establishes a versatile platform for exploring non-Hermitian magnon-polariton physics, where the engineered manipulation of magnon dissipation is essential for advanced magnonic functionalities.

## Supplementary Information

Below is the link to the electronic supplementary material.

Supplementary Material

## Data Availability

All raw data corresponding to the results in this manuscript are available on GitHub (https://github.com/Shugo0117/uno-paper2025/tree/v1.0) and archived at Zenodo (10.5281/zenodo.17427760).
