# Supplementary material for: Tunable magnon polaritons via Eddy-current-induced dissipation in metallic-banded YIG spheres
Source: Sci Rep. 2026 Aug 19;16:26018. doi: 10.1038/s41598-026-55914-x (PMC13486674; doi:10.1038/s41598-026-55914-x)
Supplement: Supplementary file 1 [file 41598_2026_55914_MOESM1_ESM.docx]

Supplemental materials for

Tunable Magnon Polaritons via Eddy-Current-Induced Dissipation

in Metallic-Banded YIG Spheres

Tatsushi Uno^1^, ^*^Shugo Yoshii^1^, Sotaro Mae^1^, ^†^Ei Shigematsu^1,3^,

Ryo Ohshima^1,3^, Yuichiro Ando^1,3,4^, and ^‡^Masashi Shiraishi^1,3^

^1^ Department of Electronic Science and Engineering, Kyoto University, Kyoto 615-8510, Japan

^2^ Institute for Chemical Research, Kyoto University, Uji 611-0011, Japan

^3^ Center for Spintronics Research Network, Kyoto University, Uji 611-0011, Japan

^4^ PRESTO, Japan Science and Technology Agency, Saitama 332-0012, Japan

^*^ [yoshii.shugo.63x@kyoto-u.jp](mailto:yoshii.shugo.63x@kyoto-u.jp)

^†^ [shigematsu.ei.2r@kyoto-u.ac.jp](mailto:shigematsu.ei.2r@kyoto-u.ac.jp)

^‡^ [shiraishi.masashi.4w@kyoto-u.ac.jp](mailto:shiraishi.masashi.4w@kyoto-u.ac.jp)

A. **Identification of FMR mode in VNA experiments**

In VNA experiments, various modes other than FMR mode are excited by the inhomogeneous AC magnetic field from CPW. To identify FMR mode, the frequency and static magnetic field of *S*_21_ spectra were normalized. In Ref. 1, the normalized frequency $\tilde{f}$ and static magnetic field $\tilde{H}$ are expressed as follows.

| $\tilde{f}=\frac{f}{\gamma\mu_{0}M}-\frac{\mu_{0}H}{\mu_{0}M}+\frac{1}{3},$ | S1(a) |
| --- | --- |
| $\tilde{H}=\frac{\mu_{0}H}{\mu_{0}M},$ | S1(b) |

where $H$, $M$, $f$ are the static magnetic field, magnetization, frequency, respectively. The |*S*_21_|^2^ spectra of the VNA experiments in the main text normalized by $\tilde{f}$ and $\tilde{H}$ are shown in Fig. S1. The spectra differ between out-of-plane and in-plane magnetic field because the VNA senses variations of YIG magnetization differently depending on the direction of the static magnetic field. Considering FMR mode is located as a straight line parallel to the x-axis at $\tilde{f}=$0 in Ref. [1] and should have the strongest spectra, FMR mode of each case in Fig S1. is identified as the mode inside the white line.

Fig. S1. |*S*_21_|^2^ spectra of each sample case in VNA measurements. The mode within the white line is the FMR mode. (a)-(c) out-of-plane magnetic field configuration and (d)-(f) in-plane magnetic field configuration. (a)(d) Sample-bare, (b)(d) Sample- SiO_2_/Cu, and (c)(f) Sample-Pt, respectively.

**B. Resonance frequency dependence of half-width at half-maximum:** $\boldsymbol{\mu}_{\boldsymbol{0}}\boldsymbol{\Delta H}$

We have analyzed the frequency dependence of the FMR linewidth for both configurations. Figures S2(a) and S2(b) show the frequency dependence of FMR peak linewidth, extracted using Eq. (6) in the main text, for the OOP and IP configurations of all the samples. For the bare YIG sphere, the linewidth increases monotonically as the frequency decreases, consistent with the low-frequency enhancement of magnon damping reported by A. Pacewicz *et al*. [2]. This enhancement is strongly suppressed near the 9 GHz frequency of the ESR cavity used in this work, explaining why the absolute linewidth values differ between the lower-frequency CPW measurements and the higher-frequency ESR measurements. This frequency dependence is intrinsic to YIG and is unrelated to the eddy-current effect.

For the OOP configuration, Sample-SiO_2_/Cu and -Pt exhibit a similar frequency dependence to the bare sample. In contrast, for the IP configuration – where eddy-current damping is dominant – the linewidth increases proportionally with the resonance frequency, in agreement with the second term of Eq. (11) in the main text. To isolate the eddy-current contribution, we subtracted the linewidth of Sample-bare from that of Sample-SiO_2_/Cu and -Pt in the IP configuration (Fig. S2 (c)). Assuming that Sample-bare linewidth corresponds to the first term in Eq. (11) in the main text, the residual linewidth should be proportional to *β*. Indeed, Fig. S2(c) shows a clear linear dependence on frequency, from which we estimate *β* ~ 9.42 × 10^-13^ for Sample-SiO_2_/Cu and *β* ~ 1.31 × 10^-12^ for Sample-Pt. These values are in good agreement with the independent estimates obtained from other measurements in the main text. Thus, the absolute difference in linewidth between CPW and ESR measurements arise from the intrinsic frequency dependence of magnon damping in YIG, while the quantitative OOP-IP trends and the extracted eddy-current parameters remain consistent across both methods.

Fig. S2 Resonance frequency dependence of linewidth for Sample-bare (red points), -SiO_2_/Cu (green points), and -Pt (blue points) for (a) OOP and (b) IP configurations. (c) Resonance frequency dependence of the residual linewidth of sample-SiO_2_/Cu and -Pt.

C. **Estimation ofβthrough the I-V measurement for Sample-SiO_2_/Cu**

Fitting was performed on the linewidths of sample-SiO_2_/Cu in Fig 2(d) by using the following equation,

| $A+\frac{\omega\beta\mu_{0}M_{z}\cos^{2}\theta}{2}\mathbf{.}$ | S2 |
| --- | --- |

Here, $A$ represents the linewidth inclusive of the influence of the Cu band at 0 degrees. The fitting results are shown in Fig 4(d) of the main text and $\beta$ was estimated to be 1.22 × 10^-12^ s/rad.

The inductive voltage $V$ in the Cu band at 0 degrees is,

| $V=-\frac{d\boldsymbol{B}\cdot\boldsymbol{S}}{dt},$ | S3 |
| --- | --- |

where $\boldsymbol{B}$ and $\boldsymbol{S}$ are the magnetic flux density and the area vector of the equatorial great circle of YIG sphere normal to its face, respectively.

$\boldsymbol{B}$ is expressed as $\mu_{0}M_{x}e^{j\omega t}$ using the precession component of the magnetization of YIG. $S$ becomes $\pi r^{2}$, where $r$ is the radius of the YIG sphere. Substituting these equations into Eq. S3 yields,

| $V=-j\pi r^{2}\omega\mu_{0}M_{x}e^{j\omega t}.$ | S4 |
| --- | --- |

The electric current $I$ flowing in the Cu band is expressed with the resistance, $R$, of the Cu band as

| $I=\frac{-j\pi r^{2}\omega\mu_{0}M_{x}e^{j\omega t}}{R}.$ | S5 |
| --- | --- |

The AC magnetic field $h_{\mathrm{eddy}}$ generated by $I$ is,

| $h_{\mathrm{eddy}}=\frac{-j\pi r\omega\mu_{0}M_{x}e^{j\omega t}}{2R}.$ | S6 |
| --- | --- |

Comparing Eq. 7(a) and Eq. S6, $\beta_{\mathrm{DC}}$ is expressed as

| $\beta_{\mathrm{DC}}=\frac{\pi r\mu_{0}}{2R}.$ | S7 |
| --- | --- |

To estimate the value of $\beta_{\mathrm{DC}}$, two-terminal resistance of the Cu band was measured. The experimental setup is shown in Fig. S3(a). Sample-SiO_2_/Cu was fixed on a SiO_2_ substrate. After creating a gap in the Cu band, the extremities of the Cu band and a current source were electrically connected by gold wire and silver paste. The voltage value was measured while varying the current value from -0.5 mA to 0.5 mA.

Fig. S3(b) shows the I-V measurement results. From the slope of the current as a function of the applied voltage, $R$ was estimated at approximately 356 Ω. Substituting $r=$0.5 mm, $\mu_{0}=$4π × 10^-7^ H/m, and $R=$356 Ω into Eq. S7 yields, $\beta_{\mathrm{DC}}=$ 2.77 × 10^-12^ s/rad. This value is comparable within the same order magnitude to what was obtained from the VNA measurement (8.0 × 10^-13^ s/rad) and ESR measurement (1.22 × 10^-12^ s/rad). More than factor-of-two differences from RF measurement can be attributed to the estimation by DC measurement of resistance excluding additional factors such as contact geometry and possible frequency-dependent conductivity. Further investigation of these factors is required for the precise estimation of $\beta_{\mathrm{DC}}$.

Fig. S3. (a) Schematic image of measurement setup of the resistance of the Cu band. Sample-SiO_2_/Cu was placed on a SiO_2_ substrate. A small part of Cu band was removed, and both edges were connected to a current source by gold wire and silver paste. (b) The result of the resistance measurement of Cu band. The solid line represents a fitting line. From the fitting, the resistance was evaluated to be 356 Ω.

**D. Angular dependence of FMR induced by coil**

To determine FMR mode in magnon-photon coupling measurement in the main text, we measured FMR with rotating sample-Pt in the set-up of using the same electronic magnet. Fig. S4 (a) shows the schematic of measurement set-up for angular dependence of FMR excited by the coil where the rutile resonator and Cu shield are removed from the set-up of Fig. 5 (a) in the main text. Here, we applied from 0 mA to 2000 mA into an electromagnet and measured reflection |*S*_11_| between 1 GHz and 4 GHz by VNA. Fig. S4 (b) and (c) show |*S*_11_| as a function of coil current into the electromagnet at 90° and 0°, respectively. They clearly show FMR mode peaks with the difference of resonance point depending on the angle of external static magnetic field against the plane of a metallic band. Considering the same relation between applied static magnetic field and coil current with magnon-photon coupling measurement set-up, the modulation of spectra shown Fig. 5 (c)-(e) in the main text means the tuning of magnon-photon coupling between Kittel mode in YIG sphere and photon mode. Fig. S4 (d) shows the magnetic angular dependence of spectra at 0 mA. The shift of resonance frequency by rotating sample-Pt can be attributed to the crystalline magnetic anisotropy of YIG sphere. Therefore, the shift of coupling point in Fig. 5 (c)-(e) by rotating the sample is induced by change of FMR resonance point due to the crystalline magnetic anisotropy of YIG sphere.

Fig. S4. (a) Schematic of magnetic angular dependence of FMR excited by coil in YIG sphere with a metallic band. FMR spectra at (b) 90° and (c) 0°, where the broken line shows the level of 2.61 GHz, the resonance frequency of rutile resonator in our measurement. (d) Magnetic angular dependence of FMR spectra extracted at 0 mA.

**E. Depth dependence of** $\boldsymbol{g}$ **and** $\boldsymbol{\kappa}_{\mathbf{m}}$ **in sample-bare and -Pt**

The depth dependence of $g$ and $\kappa_{m}$ in sample-bare and -Pt was measured to investigate the variations of the parameters which can be fluctuated by the positions of the samples. The depth of the samples was defined as shown in Fig. S5(a). The position where the samples just inside the cavity is defined as 0 mm. In the case of sample-Pt, the rotation angle was set to 90 degrees. The voltage reflection *S*_11_ spectra of sample-bare and -Pt were measured at 0.25 mm intervals from -0.5 mm to 1.5 mm depth. The power and the ranges of frequency and the applied magnetic field were the same as those in the main text. We note that the photon-magnon measurements of sample-Pt in the main text were performed at the depth of 1 mm.

Fig. S5 (b)~(f) ((g)~(i)) show the 2Dmaps of *S*_11_ at each depth of sample-bare (sample-Pt). The depth is indicated in the bottom right corner of the figures. In both samples, the split of the anti-crossing levels becomes larger as the depth increases. $g$ and $\kappa_{m}$ of each measurement evaluated by Eq. (12) are shown in Fig. S5(j) and (k). Fig. S5(j) shows that the behavior of $g$ does not change in both cases with or without a metal band. From Fig. S5(k), it is indicated that $\kappa_{m}$ of YIG remarkably increases when it is equipped with a metal band, and that there is no significant fluctuation which cancels the above systemically increase by artifacts when setting up the samples position depth.

It is worth discussing power dependence of magnon damping induced by metallic band. As the rf power increases, the cone angle of the magnetization precession increases, which results in the enhancement of the x-component in magnetization *M*_x_. Certainly, this enhancement leads to an increase of induced eddy current in the metallic band. However, in the present study, we evaluate the magnon dynamics of the metal-banded YIG system through the susceptibility spectra, where the response is normalized by the amplitude of the driving microwave magnetic field rather than by the bare output signal. Under the assumption that *M*_x_, *M*_y_ << *M*_z_ in the linear regime of typical magnetization precession, the susceptibility is not dependent on *M*_x_ or *M*_y_ as derived in Eq. (8) in the main text.

We can qualitatively simulate the power-dependence of magnon damping *κ*_m_ from the results of depth dependence of YIG sphere since the magnitude of rf magnetic field monotonically increases as the YIG sphere is placed closer to the center of the rutile resonator, as can be seen in Fig.1 (c) of the paper in Ref. 5, where a rutile resonator with the same design was used. Fig. S5 (l) shows the deviation of *κ*_m_ between Sample-bare and Sample-Pt, extracting the contribution of metallic band to *κ*_m_. Although it shows the slight enhancement of *κ*_m_ around 1 MHz, the increase of *κ*_m_ is not significant compared with the change induced by varying the angle of external magnetic field (~ 65 MHz).

Fig. S5. (a) Schematic image of the cavity and the sample setup. Definition of the sample depth is also shown. The depth is measured from the position when the right end of sample is located at the cavity edge. (b)-(e) Two dimensional maps of |*S*_11_/$S_{11}^{\mathrm{BG}}$| of sample-bare at each depth. The depth is shown in inset of the figures. (f)-(i) Two dimensional maps of |*S*_11_/$S_{11}^{\mathrm{BG}}$| of sample-Pt at each depth. (j) Depth dependence of $g$. (k) Depth dependence of $\kappa_{m}$. The above two parameters are extracted from the |*S*_11_/$S_{11}^{\mathrm{BG}}$| spectra. (l) Deviation of *κ*_m_ between Sample-bare ($\kappa_{m}^{\mathrm{Bare}}$) and Sample-Pt ($\kappa_{m}^{\mathrm{Pt}}$) as a function of depth of YIG sphere.

**F. Angular dependence of** $\boldsymbol{g}$ **and** $\boldsymbol{\kappa}_{\mathbf{m}}$ **in sample-bare**

The angular dependence of $g$ and $\kappa_{m}$ of sample-bare was measured. The depth was fixed at 1 mm (the zero definition is shown in Fig. S5(a)). The power and frequency of microwave and the ranges of the applied magnetic field were the same as those described in the main text. The 2Dmaps of *S*_11_ were obtained from 90 degrees to 90 degrees in 30 degrees increments. Fig. S6(a)~(d) show the 2Dmap of *S*_11_ of each angle. The angular dependence of $g$ and $\kappa_{m}$ are illustrated in Fig. S6(e). It is indicated that $g$ and $\kappa_{m}$ remain constant with respect to the angle and that the sinusoidal angular dependence of $\kappa_{m}$in sample-Pt is induced due to the Pt band.

Fig. S6. (a)-(d) Two dimensional maps of |*S*_11_| of sample-bare at each angle. The artifactual background changes during the measurement can be seen in (b) and (d), but it does not affect the evaluation of the parameters. (e) Angular dependence of $g$ (red circles) and $\kappa_{m}$ (blue circles) of Sample-bare. $g$ and $\gamma$ are constant with respect to angle increment.

**G. Direct inductive coupling between CPW and the metallic loop band**

The metallic band can be directly coupled to the rf magnetic field generated by the CPW. In the setup design, which was designed to maximize the eddy current induced by magnetization precession, the dominant rf magnetic field generated by the CPW is approximately parallel to the metal band plane. Nevertheless, a small magnetic-field component normal to the loop plane can arise from slight misalignment and field nonuniformity, and this component may induce an additional loop current. In fact, the loop current should generally be written as the response to both the CPW-generated flux (small unintended y-component of $h_{\mathrm{CPW}}$) and the flux generated by the magnetization dynamics:

| $I_{\mathrm{loop}}=-\frac{j\omega}{R}\left( \mu_{0}\int_{\mathrm{loop}} h_{\mathrm{CPW}}^{y}dS+\mu_{0}A_{c}M_{y} \right)e^{j\omega t},$ | S8 |
| --- | --- |

where $A_{c}=\pi r^{2}$, $R$, $r$, and $\omega$ are the area of the inside of the metal band loop, the resistance of the metallic loop, the radius of the YIG sphere, and the angular frequency of the rf magnetic field, respectively. Here, we use the *xyz* coordinates defined in the CPW experiment’s setup (Fig. 2(a) in the main text). The first term represents the direct CPW-loop coupling, whereas the second term corresponds with the magnetization-induced eddy current responsible for the additional magnon damping in YIG sphere.

Assuming the $h_{\mathrm{CPW}}^{y}$ is approximately uniform over the loop area, the rf field generated by this loop current can be written as

| $h_{\mathrm{loop}}^{y}=-\frac{j\omega}{2rR}\left( \mu_{0}A_{c}h_{\mathrm{CPW}}^{y}+\mu_{0}A_{c}M_{y} \right)e^{j\omega t}.$ | S9 |
| --- | --- |

We note that the first term, $\mu_{0}A_{c}h_{\mathrm{CPW}}^{y}$, is expected to be much smaller than the second term, $\mu_{0}A_{c}M_{y}$, due to the huge $M_{y}$ susceptibility, $\frac{M_{y}}{h_{\mathrm{CPW}}^{x}}$ , together with the geometrical relation: $h_{\mathrm{CPW}}^{y}\ll h_{\mathrm{CPW}}^{x}$. Thus, the loop current by $h_{\mathrm{CPW}}^{y}$ is negligible in the CPW system. In addition, in the ESR and rutile cavity systems, the coordinates are chosen so that the well-defined AC magnetic field is parallel to the metallic band face, which further suppresses the component normal to the loop plane.

**Reference**

[1] P. C. Fletcher and R. O. Bell, “Ferrimagnetic resonance modes in spheres”, J. Appl. Phys. **30**, 687 (1959).

[2] A. Pacewicz, J. Krupka, B. Salski, P. Aleshkevych, and P. Kopyt, “Rigorous Broadband study of the intrinsic ferromagnetic linewidth of monocrystalline garnet spheres”, Sci. Rep. **9**, 9434 (2019).

[3] Y. Tabuchi, S. Ishino, T. Ishikawa, R. Yamazaki, K. Usami, and Y. Nakamura, “Hybridizing Ferromagnetic Magnons and Microwave Photons in the Quantum Limit”, Phys. Rev. Lett. **113**, 083603 (2014).

[4] R. Hisatomi, A. Osada, Y. Tabuchi, T. Ishikawa, A. Noguchi, R. Yamazaki, K. Usami, and Y. Nakamura, “Bidirectional conversion between microwave and light via ferromagnetic magnons”, Phys. Rev. B **93**, 174427 (2016).

[5] K. Kato, R. Sasaki, K. Matsuura, K. Usami, and Y. Nakamura, High-cooperativity cavity magnon-polariton using a high- *Q* dielectric resonator, J Appl. Phys. **134**, 083901 (2023).
